# Impaired autophagy with augmented apoptosis in a Th1/Th2-imbalanced placental micromilieu is associated with spontaneous preterm birth

**DOI:** 10.3389/fmolb.2022.897228

**Published:** 2022-08-26

**Authors:** Khondoker M. Akram, Lucy I. Frost, Dilly OC. Anumba

**Affiliations:** Academic Unit of Reproductive and Developmental Medicine, Department of Oncology and Metabolism, The University of Sheffield, Sheffield, United Kingdom

**Keywords:** preterm birth, placental apoptosis, autophagy, Th1/Th2 immune responses, trophoblast dysfunction

## Abstract

**Background:** Despite decades of research, the pathogenesis of spontaneous preterm birth (PTB) remains largely unknown. Limited currently available data on PTB pathogenesis are based on rodent models, which do not accurately reflect the complexity of the human placenta across gestation. While much study has focused on placental infection and inflammation associated with PTB, two key potentially important cellular events in the placenta—apoptosis and autophagy—remained less explored. Understanding the role of these processes in the human placenta may unravel currently ill-understood processes in the pathomechanism of PTB.

**Methods:** To address this necessity, we conducted qRT-PCR and ELISA assays on placental villous tissue from 20 spontaneous preterm and 20 term deliveries, to assess the inter-relationships between inflammation, apoptosis, and autophagy in villous tissue in order to clarify their roles in the pathogenesis of PTB.

**Results:** We found disrupted balance between pro-apoptotic BAX and anti-apoptotic BCL2 gene/protein expression in preterm placenta, which was associated with significant reduction of BCL2 and increase of BAX proteins along with upregulation of active CASP3 and CASP8 suggesting augmented apoptosis in PTB. In addition, we detected impaired autophagy in the same samples, evidenced by significant accumulation of autophagosome cargo protein p62/SQSTM1 in the preterm villous placentas, which was associated with simultaneous downregulation of an essential autophagy gene *ATG7* and upregulation of Ca^2+^-activated cysteine protease CAPN1. Placental aggregation of p62 was inversely correlated with newborn birth weight, suggesting a potential link between placental autophagy impairment and fetal development. These two aberrations were detected in a micromilieu where the genes of the Th2 cytokines *IL10* and *IL13* were downregulated, suggesting an alteration in the Th1/Th2 immune balance in the preterm placenta.

**Conclusion:** Taken together, our observations suggest that impaired autophagy and augmented apoptosis in a Th1/Th2 imbalanced placental micro-environment may be associated with the pathogenesis of spontaneous PTB.

## Introduction

Parturition before 37 gestational weeks, defined as preterm birth (PTB), is the leading cause of deaths in children under five. Every year ∼15 million babies are born preterm globally and over one million of them die due to prematurity-associated complications (Paquette et al., 2018; Brockway et al., 2019; Chawanpaiboon et al., 2019). About 65–70% of PTB occur spontaneously and 30–35% are medically indicated to mitigate maternal and fetal complications (Goldenberg et al., 2008; Myatt et al., 2012; Eidem et al., 2015). The triggers and the mechanisms of spontaneous PTB remain poorly understood.

Approximately 30% of spontaneous PTB is associated with intrauterine infections, predominantly localised to the amnion and amniotic cavity (Goldenberg et al., 2000; Romero et al., 2001; Goldenberg et al., 2008; Romero et al., 2014), whereas, in majority of cases (∼70%) the aetiology of spontaneous PTB is unknown, hence termed idiopathic spontaneous PTB (isPTB). Despite decades of research, the pathomechanism of isPTB remains elusive. However, aberrant placental inflammation is thought to be involved. T helper 2 cell (Th2) predominant inflammation, mediated by interleukin (IL)4, IL5, IL6, IL10 and IL13 is essential to maintain gestation. At term, the Th2 inflammation switches to Th1 predominant inflammation, mediated by pro-inflammatory cytokines tumour necrosis factor-α (TNF-ɑ), interferon-γ (IFN-γ), IL2 and IL1β prior to labour (Mosmann & Coffman, 1989; Wegmann et al., 1993; Rao & Avni, 2000; Wilczyński, 2005). Premature switch from Th2 to Th1 type immune responses has been implicated in spontaneous PTB. Upregulation of Th1 cytokines IL2 and IFN-γ, and downregulation of Th2 cytokines, IL4 and IL10 in villous placenta have been reported in spontaneous PTB (El-Shazly et al., 2004).

Although several studies have explored the roles of infection and inflammation, particularly within the chorioamnion and decidua, in preterm labour, little is known about the involvement of two other important cellular processes - autophagy and apoptosis - in the pathogenesis of PTB (Maiuri et al., 2007; Nakashima et al., 2019). From placentation to parturition, the placenta undergoes continuous remodelling which requires balanced autophagy and apoptosis processes. Autophagy is an evolutionary conserved lysosomal degradation pathway driven by the sequestration of cytoplasmic contents through the formation of double-membrane vesicles, mediated by a core set of 16–20 autophagy-related genes (ATG) (Klionsky et al., 2016; Levine & Kroemer, 2019). Autophagy is essential for survival, differentiation, development, and homeostasis (Pattingre et al., 2005; Levine & Kroemer, 2008). A basal level of autophagy is required for cellular homeostasis which involves the removal of intrinsic toxins, damaged organelles and protein aggregates (Levine & Kroemer, 2008, 2019; Mariño et al., 2011).

Autophagy plays crucial roles in trophoblast functions and vascular remodelling during normal placental development (Arikawa et al., 2012; Nakashima et al., 2013; Arikawa et al., 2016). Lack of ATG7 in villous trophoblasts abrogates normal placental development (Aoki et al., 2018) and reduces fetal birth weight in mice (Muralimanoharan et al., 2016). Accumulation of the autophagosome cargo protein p62 (NUP62), also known as sequestosome 1 (SQSTM1) (Bloemberg & Quadrilatero, 2019), in the extravillous trophoblast cells in preeclamptic placentas has been detected, suggesting inhibition of autophagy (Nakashima et al., 2013; Nakabayashi et al., 2016). There is a lack of evidence that dysregulated autophagy is involved in spontaneous PTB in humans. However, defective autophagy has been shown to result in the accumulation of aberrant proteins that impair placental development through induction of cellular senescence and apoptosis (Nakashima et al., 2019).

Apoptosis is a physiological process of cell death, essential for normal tissue homeostasis. It is centrally regulated by the BCL2 family proteins, including pro-apoptotic proteins BAX, BAK and BAD, and anti-apoptotic proteins BCL2, BCL-XL (BCL2L1), BCL-W (BCL2L2) and MCL1. The pro-apoptotic BAX promotes apoptosis via mitochondrial release of cytochrome C, and the anti-apoptotic BCL2 inhibits apoptosis by inhibiting BAX activity (Yang & Korsmeyer, 1996; Wickremasinghe & Hoffbrand, 1999). Aberrant apoptosis in trophoblasts leads to adverse pregnancy outcomes such as intrauterine growth restriction (Barrio et al., 2004; Endo et al., 2005) and preeclampsia (Allaire et al., 2000) in animals but there is limited data on how placental apoptosis plays a role in the pathogenesis of PTB. Upregulation of the *BAX* gene (Demendi et al., 2012) and altered BAX/BCL2 protein ratio with downregulated BCL2 were demonstrated in preterm placenta, suggesting a potential role for increased apoptosis in the pathogenesis of PTB (Daher et al., 2008).

Inflammation, autophagy and apoptosis are interlinked (Pattingre et al., 2005; Su et al., 2013; Mariño et al., 2014; Cadwell, 2016). Autophagy and apoptosis frequently occur in the same cell, following a sequence where autophagy precedes apoptosis (Maiuri et al., 2007). While pro-inflammatory cytokines, such as TNF-α (TNF), induce apoptosis (Rath & Aggarwal, 1999; Wang et al., 2008), the anti-inflammatory cytokine IL10 inhibits it by upregulating the anti-apoptotic gene *BCL2* (Levy & Brouet, 1994; Dhingra et al., 2011). BCL2 is also a negative regulator of a key autophagic protein Beclin 1 (BECN1) and inhibits autophagy (Pattingre et al., 2005; Mariño et al., 2014). Under stressful conditions, augmented apoptosis inactivates autophagy by caspase-mediated cleavage of key autophagy mediators (Luo & Rubinsztein, 2010; Wirawan et al., 2010; Oral et al., 2012).

How the ‘inflammation-autophagy-apoptosis’ axis altogether is modulated in the human term and preterm placenta has not been studied. To understand this interplay, we conducted a series of qRT-PCR and ELISA assays on spontaneously delivered term (n = 20) and preterm (n = 20) fresh human placentas to evaluate the molecular signatures of apoptosis, autophagy (macroautophagy) and inflammation in the villous micro-environment within the same placental cotyledon of each placenta. We found augmented apoptosis with impaired autophagy in a pro-inflammatory micro-environment of preterm villous placenta, suggesting a mechanistic link of dysregulated inflammation-autophagy-apoptosis axis in the pathogenesis of spontaneous PTB.

## Materials and Methods

### Participant recruitment

From April 2019 to December 2020, a total of forty pregnant women with age >16 years who delivered at the Jessop Wing Maternity Hospital, Sheffield, United Kingdom were recruited for the study (Table 1). Amongst them, 20 women delivered at term (>37 weeks of gestation) and 20 women delivered at preterm (<37 weeks of gestation). Women carrying singleton pregnancies and initiated labour spontaneously without having any urinary and genital tract infections were included in this study.

**TABLE 1:** The study participants criteria. Data is presented as median with ranges in the parentheses. *p* values are calculated by either Mann-Whitney *U* test or ^#^Fisher’s exact test.

Participant criteria	Term	Preterm	*p* Values
	n = 20	n = 20	
Gestational age at delivery, wks (Range)	**39.6** (37.4–41.1)	**33.5** (24–36.6)	<0.0001
**Subtypes of preterm births (%)**			
Extremely preterm (<28 weeks)		2 (10%)	
Very preterm (28–31 weeks)		4 (20%)	
Moderate preterm (32–33 weeks)		5 (25%)	
Late preterm (34 - < 37 weeks)		9 (45%)	
Spontaneous labour	100%	100%	
**Membrane rupture status (%)**			
SROM	12 (60%)	1 (5%)	<0.0001^#^
PPROM	0 (0%)	10 (50%)	
AROM	7 (35%)	8 (40%)	
Others	1 (5%)	1 (5%)	
Maternal age at delivery, yrs (Range)	**31.1** (22.2–40.1)	**27.5** (17.8–40.7)	0.0924
**Race, n, (%)**			
White	17 (85%)	18 (90%)	
Black	2 (10%)	0 (0%)	
Asian and others	1 (5%)	2 (10%)	
BMI at delivery, kg/m^2^ (Range)	**27.1** (21.2–44.3)	**27.5** (17.7–53)	0.6902
Preexisting diabetes mellitus	0	0	
**Smoking (%)**			
Yes	3 (15%)	4 (20%)	
No	17 (85%)	16 (80%)	
**Mode of delivery (%)**			
Vaginal (VD)	17 (85%)	14 (70%)	0.3377^#^
Caesarean (CS)	1 (5%)	3 (15%)	
Instrumental	2 (10%)	3 (15%)	
Baby birth weight, kg (Range)	**3.5** (2.3–4)	**1.7** (0.6–3)	<0.0001
Placenta gross weight, grams (Range)	**674** (490–897.2)	**409** (192–679.3)	<0.0001

SROM, spontaneous rupture of membranes; PPROM, preterm premature rupture of the membranes; AROM, artificial rupture of membranes.

### Placenta tissue samples

The placentas were collected, and the tissue samples were processed within 3 h of delivery. Tissue samples were harvested in a biosafety level-2 laminar flow hood under sterile condition. For qRT-PCR (Real-Time Quantitative Reverse-Transcription Polymerase Chain Reaction), a 2 cm by 2 cm tissue flap of the decidua basalis was removed from an intact lobe of the maternal surface of the placenta. Approximately 1 gm of villous tissue (devoid of decidua) was excised and washed twice in sterile PBS (ThermoFisher, Cat. No- 10010056) to remove intervillous maternal blood, and transferred into a 2 ml cryovial (Corning, Cat. No-CLS430659) containing 1.5 ml of RNA Later buffer (Qiagen, Cat. No- 76106) and left to stand for 30 min at room temperature before storing it in the −80°C freezer until further processing. For ELISA assay, a block of villous tissue (1 cm^3^) was taken from the same lobe where the tissue for qRT-PCR was taken, and immediately snap-frozen in a cryovial for 1 h in liquid nitrogen before storing it in the −80°C freezer.

### RNA extraction

Total RNA was extracted from the villous tissue samples using RNeasy Plus Mini Kit (QIAgen, Cat No- 74134), following the manufacturer’s instruction. Briefly, the frozen samples were thawed at room temperature, and 30–35 mg of tissue was taken from each sample in RLT Lysis buffer supplemented with β-mercaptoethanol (Sigma, Cat no- M3148) into a sample shredder tube (Qiagen, Cat No- 990381) along with a 5 mm diameter stainless steel bead (Qiagen, Cat No- 69989). Tissue was completely homogenised using a TissueLyser LT homogeniser (Qiagen, Cat No- 85600) for 10 min at 50 Hz speed. Tissue homogenate was centrifuged for 3 min at 18,000 x g and the supernatant was filtered through a gDNA eliminator spin column to remove genomic DNA. Total RNA was extracted using a mini spin column as per the manufacturer’s protocol. Purified total RNA was eluted in 50 µL of RNase-free water. The quality and quantity of the yield RNA was checked using Nanodrop ND1000 and Qubit RNA HS Assay Kit (ThermoFisher, Cat No- Q32852). RNA samples were stored in -80°C.

### qRT-PCR

One-step qRT-PCR was conducted using 100 ng of total RNA from each sample using QuantiNova SYBR Green RT-PCR Kit (Qiagen, Cat. No- 208154) and QuantiTect Primer Assay primer probes (Qiagen, Cat. No- 249900) (Supplementary Table S1) following the manufacturer’s instruction. The qRT-PCR reactions were done in duplicates for each sample on a Rotor-Gene Q (Qiagen, Cat No- 9001550) following the thermocycling protocol as follows: reverse transcription at 50°C for 10 min followed by inactivation at 95°C for 2 min, then 40 cycles of PCR with denaturation at 95°C for 5 s and combined annealing/extension at 60°C for 10 s for each cycle. A melting curve analysis was performed for each qRT-PCR run. The melting curve for each primer confirmed specificity of each primer used in this study. GAPDH was used as a housekeeping gene for normalisation of data which was expressed equally across all 40 samples (Supplementary Figure S1). The Ct values were used for calculation of relative gene expression (2^−ΔCt^, relative to GAPDH) in term and preterm groups independently or as gene expression fold-changes in preterm compared to the term group (2^-∆∆Ct^) (Livak & Schmittgen, 2001; Saaed et al., 2017). Data were presented as Log_10_ relative gene expression.

### Protein extraction and ELISA assay

Total protein was extracted from the snap-frozen villous tissue using SigmaFAST/IgePal lysis buffer following the manufacturer’s instructions. Briefly, 100 mg of villous tissue was taken in 1 ml of SigmaFAST/IgePal lysis buffer, made up of 0.1% IgePal CA-630 (Sigma-Aldrich, Cat No- I3021) with SigmaFAST Protease Inhibitor Cocktail (Sigma-Aldrich, Cat No- S8830) (1 tablet dissolved in 100 ml PBS) in PBS in a sample shredder tube along with a metal bead as described above. Tissue was homogenised by TissueLyser LT for 10 min at 50 Hz at room temperature followed by an incubation at 4°C for 30 min on a horizontal roller. After incubation the sample was further homogenised for 5 min. Completely lysed tissue homogenate was centrifuged at 16,000 x g for 20 min at 4°C. The supernatant was collected, and total protein concentration was determined by Qubit Protein Assay Kit (Thermo Fisher Scientific, Cat No- Q33211) following the manufacturer’s instruction. The extracted protein samples were stored at −80°C until used for ELISA assays. For ELISA assay, total protein was diluted at a concentration of 300 μg/ml in PBS for each sample. For quantification of target proteins Human BAX ELISA Kit (Fine Test, Cat No- EH0669), Human BCL2 ELISA Kit (Fine Test, Cat No- EH0658), Human p62/SQSTM1 (Sequestosome-1) ELISA Kit (Fine Test, Cat No- EH10842), Human CAPN1 (Calpain 1) ELISA Kit (Fine Test, Cat No- EH2761), Caspase 3 (Cleaved) Human ELISA Kit (Thermo Fisher, Cat No- KHO1091) and Human Cleaved CASP8 ELISA Kit (Fine Test, Cat No- EH4218) were used following the manufacturer’s instructions. A sigmoidal four-parameter logistic curve was used to generate standard curve for each quantification using GraphPad Prism (version 9.1.1).

### Statistical analysis

The data were analysed using GraphPad Prism (version 9.1.1). To calculate statistical significance the Mann-Whitney *U* test was conducted between two groups and Two-way ANOVA with Tukey’s post hoc analysis between three or more groups were conducted. A *p* value <0.05 was considered as statistically significant. Data are presented as median and interquartile ranges (IQR) with minima and maxima or as stated in the figure legends throughout the manuscript.

## Results

### Participant criteria

We recruited a total of 40 pregnant women, 20 of whom delivered spontaneously at term and 20 of whom delivered preterm, at a median gestational age (GA) of 39.6 and 33.5 weeks, respectively (*p* < 0.0001) (Table 1). Demographic details and birth outcomes are shown in Table 1. Both groups showed no clinical or bacteriological evidence of genital tract infection. Majority of women were of the Caucasian race (85% term and 90% preterm). None of the participants had diabetes mellites or hypertension. Maternal age, BMI and smoking history did not differ between the term and preterm groups. Amongst the preterm group, 45% were late preterm (34—< 37 weeks) whilst 30% were extremely (<28 weeks) or very preterm (28–31 weeks). The median newborn baby weights in preterm and term births were 1.7 vs 3.5 kg respectively (*p* < 0.0001).

### Relative reduction of *BCL2* gene expression alters BAX/BCL2 balance in preterm placenta

**FIGURE 1:**
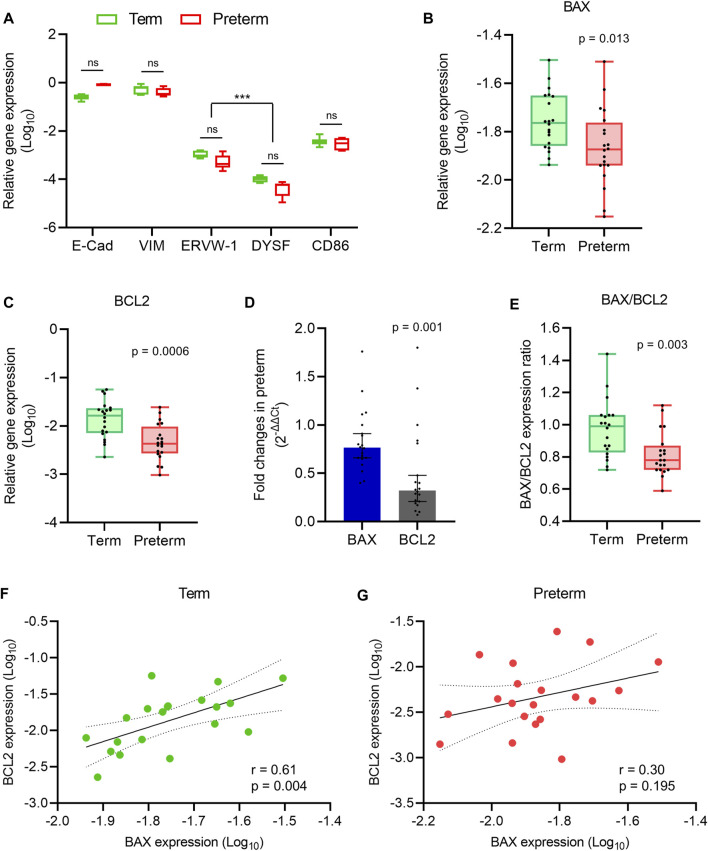
Apoptosis gene expression analysis. **(A)** Box plot of qRT-PCR analysis showing various cell-specific gene expressions (relative to *GAPDH*) in villous tissue of term and preterm placentas (n = 6 term and six preterm); ***p < 0.001; Two-way ANOVA with Tukey’s post hoc analysis. (B,C) Box plots of qRT-PCR analysis showing gene expression (relative to *GAPDH*) of *BAX* **(B)** and *BCL2* **(C)** in term and preterm placentas. **(D)** Fold-changes (Relative to term, 2^-∆∆Ct^) in the *BAX* and *BCL2* gene expression in preterm placenta compared to term placenta. **(E)** Ratio between *BAX* and *BCL2* gene expression in term and preterm placentas. **(F,G)** Spearman Rank test correlation analysis between *BAX* and *BCL2* gene expression in the term **(F)** and preterm **(G)** placentas. Dotted lines represent 95% CI bands of the best-fit lines (Solid). n = 20 term and 20 preterm **(B–G)**; ns = not significant. Mann-Whitney *U* test **(B–E)**. Box plots data are presented as median and interquartile ranges (IQR) with minima and maxima. Each dot indicates an individual subject **(B–G)**.

Before evaluation of *BAX* and *BCL2* gene expression, we identified the major cell types present in the villous tissue by using cell-specific gene markers. The villous tissue consists of various cell types, including cytotrophoblasts, syncytiotrophoblasts, mesenchymal cells, endothelial cells and immune cells, predominantly placental macrophages of fetal origin (Hofbauer cells) (Vinnars et al., 2010; Pereira, 2018; Turco & Moffett, 2019; Swieboda et al., 2020). Our qRT-PCR analysis identified the presence of epithelial-like cells (*E-Cadherin (CDH1)*, expressed by trophoblasts) and mesenchymal cells (Vimentin) in high abundance as seen previously (Suryawanshi et al., 2018) (Figure 1A). We detected *CD86* gene in villous tissue which is expressed by a wide variety of immune cells including macrophages (Vinnars et al., 2010; Swieboda et al., 2020). The presence of cytotrophoblasts and syncytiotrophoblasts were detected by *ERVW-1* (a marker for cytotrophoblasts (also known as Syncytin-1)) and Dysferlin (*DYSF*) (a marker for syncytiotrophoblasts) expression (Vandré et al., 2007; Suryawanshi et al., 2018) (Figure 1A). The expression levels of the aforementioned genes were not significantly different between the term and preterm placentas, indicating a similar distribution of these cell types in both groups (Figure 1A).

Next, to assess the level of apoptotic signalling, we conducted qRT-PCR on villous tissue harvested from the 20 term and 20 preterm fresh placentas to quantify the expression of the pro-apoptotic gene *BAX*, and the anti-apoptotic gene *BCL2* (Kazi et al., 2011). Both *BAX* and *BCL2* gene expressions were significantly decreased in the preterm placentas compared to the term placentas (*p* = 0.013 and *p* = 0.0006, respectively; Figures 1B,C). However, the *BCL2* gene expression fold-change was 58% lower than the *BAX* expression in preterm placentas (0.32-fold vs. 0.76-fold, respectively, 95% CI = - 0.594 to - 0.087, *p* = 0.001; Figure 1D). Apoptosis is predominantly regulated by a critical counteracting balance between BAX and BCL2 to maintain tissue homeostasis (Kazi et al., 2011). We found an equilibrium state of *BAX* and *BCL2* gene expression (mRNA level) with a ratio of ∼1 (median = 0.99) in term placentas; which, however, was significantly altered in preterm placentas (median = 0.78, *p* = 0.003) due to the relatively higher reduction of *BCL2* expression compared to *BAX* in the preterm group (Figure 1E).

In addition, our Spearman’s correlation analysis on the same data set detected a significant positive correlation between *BAX* and *BCL2* gene expression in the term placentas (r = 0.60, *p* = 0.004; Figure 1F). Surprisingly, this BAX/BCL2 correlation was obliterated in the preterm placentas (r = 0.30, *p* = 0.195; Figure 1G). Taken together, our data demonstrated a balanced expression of *BAX* and *BCL2* genes in normal term placenta which was altered in preterm placenta due to significant relative reduction of *BCL2* expression.

### Reduced BCL2 and increased BAX protein expressions were associated with augmented apoptosis in preterm placenta

**FIGURE 2:**
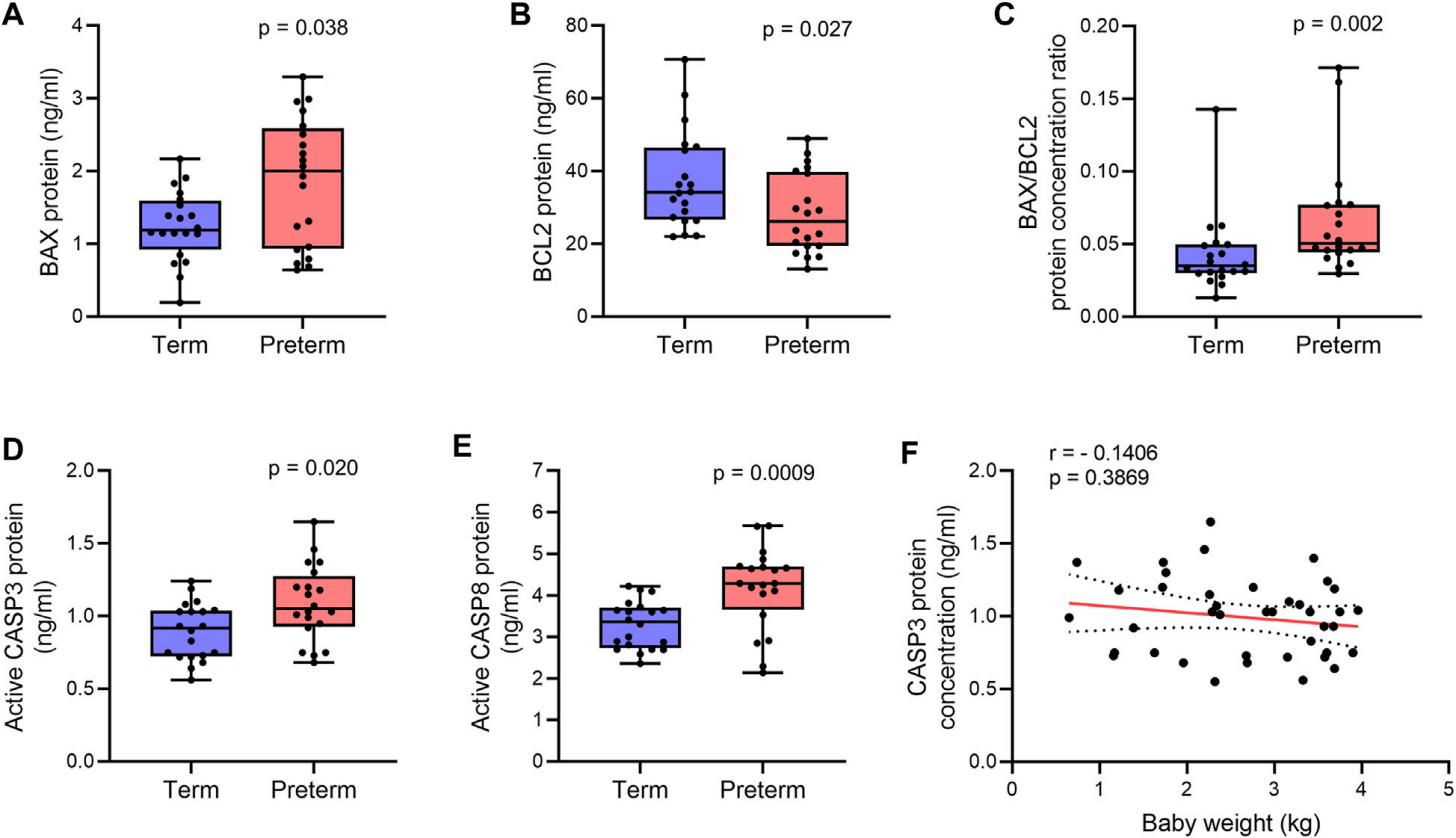
Quantification of apoptotic proteins. **(A,B)** Box plots of ELISA assays for BAX **(A)** and BCL2 **(B)** showing protein concentrations in term and preterm villous placenta tissue lysates. **(C)** Box plot showing ratio between BAX and BCL2 protein concentrations in term and preterm placentas. **(D,E)** Box plots of ELISA assays showing active CASP3 **(D)** and CASP8 **(E)** concentrations in term and preterm placentas. Total protein load was at 300 μg/ml for each sample. n = 20 term and 20 preterm **(A–E)**. Data are presented as median and IQR with minima and maxima. Mann-Whitney *U* test. **(F).** Spearman Rank test correlation analysis between CASP3 protein concentrations and baby birth weights on the entire cohort (n = 40). Dotted lines represent 95% CI bands of the best-fit lines (Solid).

We hypothesised that alteration of BAX/BCL2 gene expression balance may potentiate apoptosis in the villous placenta of PTB. To test this hypothesis, first, we quantified the levels of BAX and BCL2 protein in the villous tissue of the 20 term and 20 preterm placentas by ELISA assays. The BCL2 protein concentration was 24% lower in preterm placentas compared to term placentas (28.35 ng/ml vs 37.21 ng/ml, respectively; *p* = 0.027; Figure 2B). Although the *BAX* gene was downregulated in preterm placentas, the BAX protein concentration was found significantly higher in preterm placentas compared to term placentas (2.0 ng/ml vs. 1.2 ng/ml respectively, *p* = 0.038; Figure 2A). As expected, the BAX/BCL2 protein concentration ratio was significantly higher in preterm placentas compared to term placentas (*p* = 0.002; Figure 2C). This alteration of the BAX/BCL2 ratio suggests potential activation of the intrinsic pathway (mitochondrial pathway) of apoptosis in the preterm placenta.

Next, to confirm apoptosis within the villous tissue, we quantified the level of active (cleaved) Caspase 3 (CASP3) in villous tissue lysates of the same cohort of samples by ELISA assay. We detected a 15% higher level of active CASP3 protein in the preterm placentas compared to term placentas, suggesting upregulated apoptosis (*p* = 0.0205, Figure 2D). To further evaluate the activation of the extrinsic pathway of apoptosis in the preterm placenta villous tissue, we quantified level of active (cleaved) Caspase 8 (CASP8). We found significantly higher concentration of cleaved CASP8 in the preterm placentas compared to term placenta (4.3 ng/ml vs. 3.4 ng/ml, respectively; *p* = 0.0009; Figure 2E). To reveal if placental apoptosis is associated with fetal growth, we performed the Spearman’s correlation analysis between CASP3 concentration and baby birth weight across the whole cohort (n = 40). We did not find any significant correlation between the level of placental CASP3 and baby birth weight (r = - 0.1406, *p* = 0.3869, Figure 2F). Together, these data suggest that BAX/BCL2 imbalance due to increased level of BAX and decreased level of BCL2 enhances apoptosis in villous placenta, which is essentially not associated with fetal growth, but may have a mechanistic link with preterm labour.

### Impaired autophagy in preterm placenta associated with downregulation of *ATG7* and upregulation of CAPN1

There is an intricate balance between apoptosis and autophagy in the maintenance of tissue homeostasis (Pattingre et al., 2005; Mariño et al., 2014). Anti-apoptotic BCL2 family proteins negatively regulate autophagy by suppressing key autophagy regulator Beclin 1 (BECN1, also known as ATG6). Perturbation of this negative regulation may augment autophagy which may lead to cell death instead of tissue homeostasis (Pattingre et al., 2005; Su et al., 2013; Mariño et al., 2014). To assess the level of autophagy signalling in the villous placenta, we quantified the expression of three canonical autophagy genes (*BECN1*, *ATG3* and *ATG7*) and the accumulation of autophagosome cargo protein p62/SQSTM1 (Bloemberg & Quadrilatero, 2019) in 20 term and 20 preterm placenta villous tissue samples. We also evaluated the level of Calpain 1 (CAPN1) protein, a ubiquitous intracellular calcium-activated cysteine protease that plays critical roles in autophagy regulation and execution of apoptosis (Russo et al., 2011; Ong et al., 2019; Liu et al., 2021).

**FIGURE 3:**
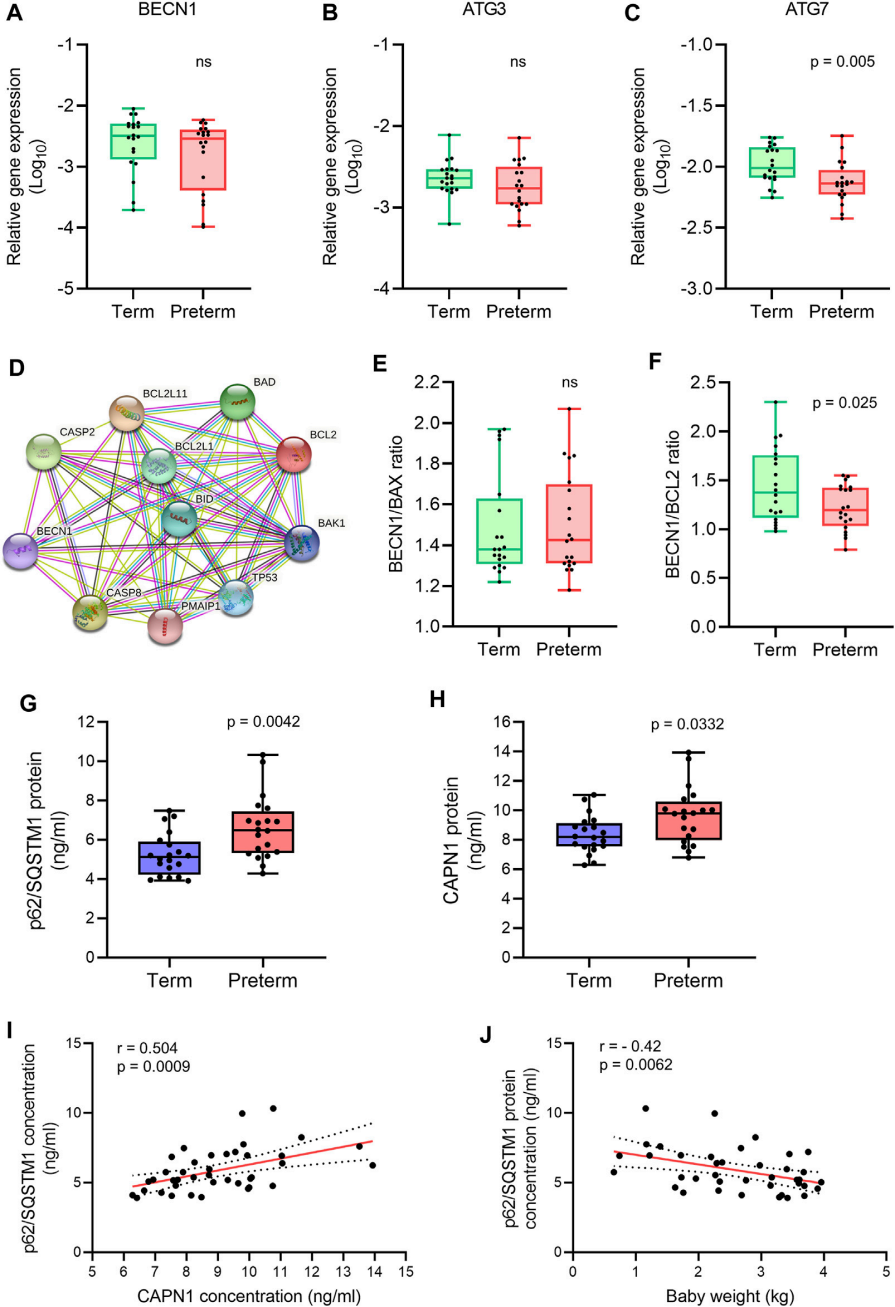
Autophagy gene and protein expression analysis. **(A-C)** Box plots of qRT-PCR analysis showing gene expression (Relative to *GAPDH*) of *BECN1*, *ATG3* and *ATG7* in term and preterm placentas. n = 20 term and 20 preterm. Data are presented as median and IQR with minima and maxima. Each dot represents individual subject. (**D)** STRING protein-protein interaction analysis (STRING, Version 11.5). **(E,F)** Ratio of gene expression of *BECN1* and *BAX* (E), and *BECN1* and *BCL2* (F) in term and preterm placentas. n = 20 term and 20 preterm. Data are presented as median and IQR with minima and maxima (**G,H).** Box plots showing ELISA assay of p62/SQSTM1 **(G)** and CAPN1 **(H)** on term and preterm villous placenta tissue lysates. Total protein load at 300 μg/ml for each sample. n = 20 term and 20 preterm. Data are presented as median and IQR with minima and maxima. **(I)** Spearman Rank test correlation analysis between p62/SQSTM1 and CAPN1 protein concentrations on the entire cohort (n = 40). J. Spearman Rank test correlation analysis between p62/SQSTM1 protein concentrations and baby birth weights on the entire cohort (n = 40). Dotted lines represent 95% CI bands of the best-fit lines (Solid). ns = not significant, Mann-Whitney *U* test (A–H).

Our qRT-PCR analysis found no significant differences in *BECN1* and *ATG3* expression in preterm placentas compared to term placentas (Figures 3A,B). However, other autophagosome-associated gene *ATG7* expression was significantly downregulated in preterm placentas compared to term placentas (*p* = 0.005; Figure 3C).

Next, we analysed the ratio between *BECN1* and its negative regulator *BCL2* gene expression in term and preterm placentas. The STRING analysis (STRING, v11.5) revealed protein-protein interactions between BECN1 with other pro- and anti-apoptotic proteins (Figure 3D). We found no significant difference of BECN1/BAX ratio between term and preterm placentas (Figure 3E). However, the BECN1/BCL2 ratio was significantly downregulated in preterm placentas compared to term placentas (*p* = 0.025; Figure 3F), suggesting a potential abrogation of negative regulatory effects of BCL2 on BECN1 in preterm placenta.

To evaluate if downregulation of *ATG7* and BECN1/BCL2 ratio modulate overall autophagy flux in villous placenta, we quantified the cytosolic level of p62/SQSTM1 protein by ELISA assay. Autophagic substrates are identified by p62/SQSTM1 protein binding to misfolded proteins which subsequently binds with LC3II facilitating autophagic degradation (Pankiv et al., 2007; Ichimura et al., 2008; Kirkin et al., 2009). Therefore, cytosolic accumulation of p62/SQSTM1 is an indication of inhibition of autophagy flux and vice versa (Bjørkøy et al., 2009; Klionsky et al., 2016). Our ELISA assay on 20 term and 20 preterm villous placentas detected a 35% higher level of p62/SQSTM1 protein in preterm placentas compared to term placenta, suggesting an inhibition of autophagy (6.9 ng/ml vs 5.1 ng/ml, respectively, *p* = 0.0042; Figure 3G), possibly attributable to downregulation of ATG7. Diminished BCL2-mediated negative regulation on BECN1 was insufficient to overcome its downstream ATG7-mediated action on net autophagy flux.

Furthermore, CAPN1 protein concentration was significantly higher in preterm placentas compared to term placentas (9.8 ng/ml vs. 8.2 ng/ml respectively, *p* = 0.033; Figure 3H). CAPN1 causes cleavage of BECN1 and ATG5 which impairs autophagy flux resulting in accumulation of autophagic substrates within the cells (Russo et al., 2011; Liu et al., 2021). Our Spearman’s correlation analysis showed a significant positive correlation between CAPN1 and p62 protein across the samples (r = 0.504, *p* = 0.0009; Figure 3I). Together, these data support the existence of impaired autophagy in preterm placenta.

To assess if accumulation of p62/SQSTM1 protein has any effect on fetal growth, we conducted the Spearman’s correlation analysis between placental p62/SQSTM1 protein concentrations and baby birth weights on both term and preterm subjects together (n = 40). Our analysis revealed a moderate but significant inverse relationship between placental p62/SQSTM1 protein and baby birth weight (r = - 0.42, 95% CI = -0.654 to -0.119, *p* = 0.0062; Figure 3J). This data suggests that inhibition of autophagy flux negatively affects fetal growth. Taken together, our data demonstrate an association of impaired autophagy with preterm birth, possibly driven by downregulation of the *ATG7* and overactivation of CAPN1.

### Alteration of Th1/Th2 inflammatory cytokine gene expression balance in preterm placenta

**FIGURE 4:**
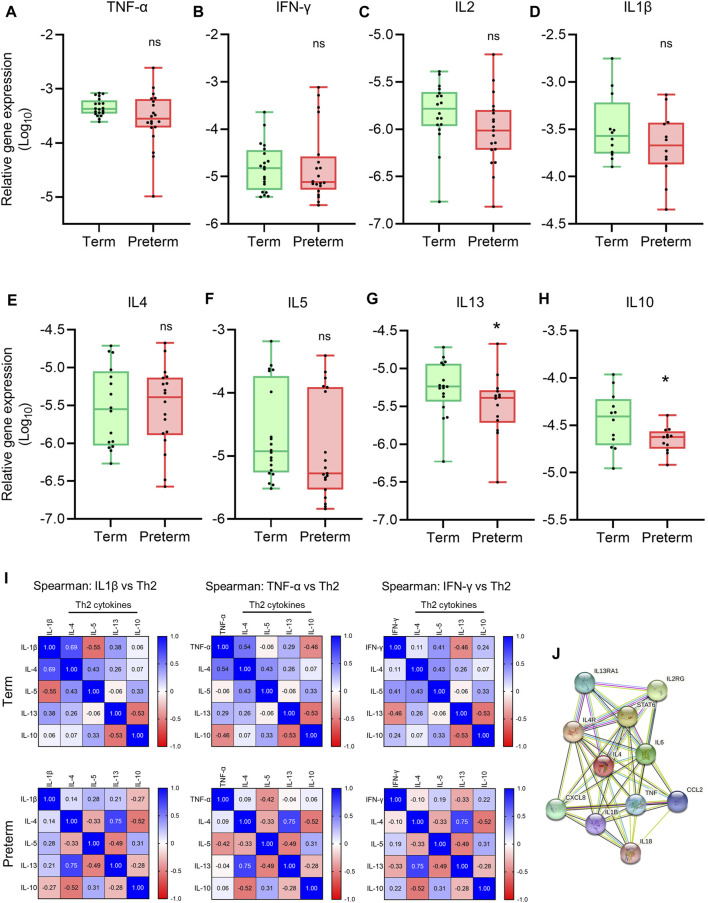
Th1 and Th2 gene expression and correlation analysis. **(A-H)** Box plots of qRT-PCR analysis showing gene expression (Relative to *GAPDH*) of Th1 cytokines **(A–D)** and Th2 cytokines **(E–H)** in term and preterm placentas. n = 20 term and 20 preterm **(A-C and E-G)**, and n = 12 term and 12 preterm **(D,H)**. Data are presented as median and IQR with minima and maxima. Each dot represents an independent subject. *p < 0.05, ns = not significant, Mann-Whitney *U* test. **(I)** Spearman Rank test correlation matrices of Th1 cytokines *IL1β*, *TNF-α* and *IFN-γ* vs Th2 cytokines *IL4*, *IL5*, *IL13* and *IL10* in term (upper panel) and preterm (lower panel) placentas. The numerical value in each small squire represents Spearman correlation coefficient ‘r’. **(J)** STRING protein-protein interaction analysis (STRING, Version 11.5).

A Th2 predominant inflammatory response is crucial to maintain pregnancy, which stitches to Th1 predominant inflammation in both maternal blood and placenta prior to labour at term (Wegmann et al., 1993; Wilczyński, 2005). The interplay between the Th1/Th2 inflammatory responses is mediated by the key Th1 cytokines TNF-ɑ, IFN-γ, IL2 and IL1β, and Th2 cytokines IL4, IL5, IL6, IL10 and IL13 (Mosmann & Coffman, 1989; Rao & Avni, 2000; Wilczyński, 2005). To evaluate the balance between Th1/Th2 inflammatory responses in placental villous tissue, we conducted qRT-PCR to quantify gene expression of a panel of Th1 and Th2 cytokines (Figure 4). We found no significant differences in gene expressions of Th1 cytokines *TNF-ɑ*, *IFN-γ*, *IL1β* and *IL2* in preterm placentas compared to term placentas (Figures 4A–D). The gene expressions of Th2 cytokines *IL4* and *IL5* were not different in preterm placenta compared to term either. However, the anti-inflammatory Th2 cytokine *IL10* gene was significantly downregulated in preterm placenta compared to term placentas (*p* = 0.04, Figure 4H). *IL13* was also significantly downregulated in preterm placentas (*p* = 0.04, Figure 4G).

To examine the correlation between pro-inflammatory Th1 cytokines TNF-ɑ, IFN-γ and IL1β, and Th2 cytokines IL4, IL5, IL10 and IL13 at the tissue level, we generated Spearman Rank test correlation matrices using gene expression data sets from the term and preterm placentas (Figure 4I). We noted a positive correlation between anti-inflammatory cytokine *IL4* gene and pro-inflammatory cytokines *IL1β* (Spearman coefficient, r = 0.69, *p* = 0.06) and *TNF-ɑ* gene expression (r = 0.54, *p* = 0.03) in term placentas (Upper panel, Figure 4I); however, these correlations were obliterated in preterm placentas (Lower panel, Figure 4I). According to the STRING analysis, *IL4* and *TNF-ɑ* are co-expressing genes (Figure 4J), which agrees with our finding in the term group. Taken together, our data provide evidence of pro-inflammatory micro-environment within the preterm placenta villous tissue due to disruption of Th1/Th2 cytokines expression, which is driven by downregulation of anti-inflammatory cytokine IL10 and abrogation for IL4/TNF-ɑ correlation.

## Discussion

The placenta is a complex organ and plays a pivotal role in the health of both fetus and mother during the entire gestation. Placental dysfunction is the primary defect in majority of adverse pregnancies, including preeclampsia, fetal growth restriction, recurrent miscarriage, stillbirth and PTB (Turco & Moffett, 2019). The main functions of the placenta are accomplished by trophoblast cells through secretion of various hormones and differentiation into cytotrophoblasts, syncytiotrophoblasts and extravillous trophoblasts (EVT). EVTs are migratory and mostly localised in the decidua (maternal part) of the placenta. In this study, we removed the decidua, washed out maternal blood and used the villous part of the placenta; therefore, our data mostly represent the fetal part of the placenta. The uniqueness of our study is that we used same villous tissue from each placenta for the simultaneous evaluation of three distinct but interlinked cellular processes: i) apoptosis, ii) autophagy and iii) inflammation, in fresh term and preterm placenta. Therefore, our data sets are linked and provide a clearer picture of the association of these three cellular events in the pathogenesis of spontaneous PTB.

**FIGURE 5:**
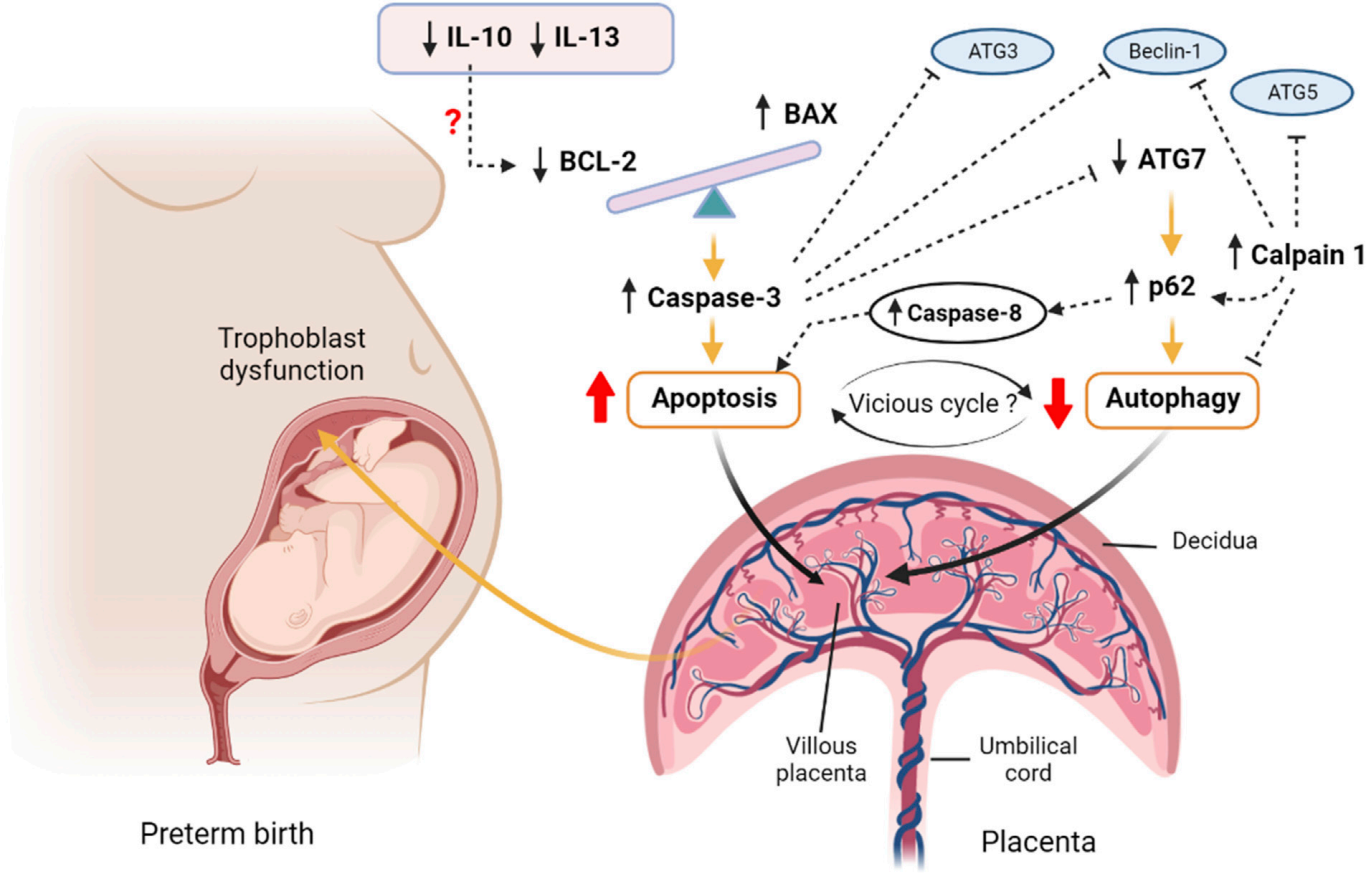
A proposed hypothesis of apoptosis and autophagy interactions in preterm birth. The imbalanced BAX/BCL2 expression in the preterm villous placenta increases the release of Caspase 3 and augments intrinsic pathway of apoptosis. The upstream downregulation of IL10 may potentiate downregulation of BCL2 expression leading to BAX/BCL2 imbalance. Caspase 8 is also activated as an indicator of potential extrinsic apoptotic pathway activation. On the other hand, the downregulation of autophagy gene *ATG7* results in accumulation of p62 cargo protein within the villous tissue which inhibits the net autophagy flux. The high level of p62 favours accumulation of effector caspases, including Caspase 8 and executes apoptosis. The aggregation of caspases including Caspase 3 degrades essential autophagy-associated proteins, such as Beclin 1, ATG3 and ATG7, which consequently inhibits autophagy. Calpain 1 cleaves ATG5 and Beclin 1 and facilitates accumulation of p62 resulting in net inhibition of autophagy flux. Thus, a vicious cycle may potentially be established between apoptosis and autophagy via the reciprocal stimulatory actions of caspases and p62 within the preterm villous placenta that may have a pathognomonic effect on PTB. The figure is created with BioRender.com.

Apoptosis is a process of physiological cell death which is essential for tissue and organ homeostasis. In placenta, apoptosis increases significantly as pregnancy progresses (Smith et al., 1997). Overexpression of pro-apoptotic gene *BAX* increases mitochondrial permeability, facilitating release of cytochrome C, which ultimately promotes the intrinsic pathway of apoptosis via activation of effector caspases, including CASP3 (Yang & Korsmeyer, 1996; Wickremasinghe & Hoffbrand, 1999; Green, 2000; Zhao et al., 2018). We detected significantly higher levels of active (cleaved) CASP3 and BAX proteins in preterm placentas compared to term placentas. At the same time, the BCL2 was downregulated at the gene (mRNA) and protein level in the preterm placentas (Figure 5). BCL2 is the key anti-apoptotic protein of the BCL2 family that directly forms a heterodimer with BAX and prevents the BAX homo-oligomerization required for mitochondrial permeabilisation to release cytochrome C, and thereby inhibits apoptosis (Wickremasinghe & Hoffbrand, 1999; Ding et al., 2010; Kazi et al., 2011). In our comparative analysis, the reduction of the mRNA level (gene expression) of *BCL2* was significantly higher than the mRNA level of *BAX* in preterm placenta. This disproportionately lower level of *BCL2* may diminish its negative regulatory effects on *BAX*, and thus enhance apoptosis (Figure 5). Furthermore, the BAX/BCL2 protein ratio was significantly higher in preterm placentas compared to term placentas suggesting a potential overactivity of BAX that may potentiate apoptosis. A previous study showed that dissociation of BAX from BCL2 heterodimers enhances apoptosis, which supports our finding (Shitashige et al., 2001). We also showed that the *BAX* and *BCL2* mRNAs were equally expressed in term placenta (ratio = ∼1), where these two regulators maintained a positive correlation, which was obliterated in preterm placenta creating an imbalanced BAX/BCL2 signalling.

A previous immunohistochemistry study on seven preterm and 25 term human placentas showed that BAX and BCL2 proteins were equally expressed in syncytiotrophoblasts and cytotrophoblasts in term placenta with a ratio of 0.93. However, the BCL2 protein expression was significantly reduced in preterm placenta compared to term placenta, whereas the BAX expression was unaltered (Daher et al., 2008). In their study, BAX/BCL2 protein ratio was increased due to downregulation of BCL2 in preterm placenta, suggesting a potential role of apoptosis in the pathogenesis of PTB. This protein expression data matches with our gene and protein expression findings (Daher et al., 2008).

However, it should be notated that, unlike the Daher et al. immunostaining finding (Daher et al., 2008), the absolute amount of BCL-2 protein fraction was higher than the BAX protein fraction in an equal amount of total protein in both term and preterm placenta, as determined by our ELISA assays. Therefore, the BAX/BCL2 ratio was not ∼1 at the protein level in term placenta as seen in case of *BAX/BCL2* ratio at the gene level. This disparity between gene and protein ratio data is unclear, however, it could be a natural phenomenon, due to relatively slower turnover of BCL2 protein compared to BAX. Furthermore, an immunostaining technique does not accurately quantify tissue protein concentration, as ELISA technique does.

Nonetheless, considering the alteration of BAX/BCL2 ratio with overactive BAX and diminished BCL2 along with increased level of active Caspase 3, we postulate a potential activation of mitochondrial (intrinsic) pathway of apoptosis in the preterm placenta (Green, 2000; Zhao et al., 2018). In addition, we noted a higher level of active Caspase 8 (CASP8) protein in preterm placenta, suggesting a potential activation of extrinsic pathway of apoptosis as well (Green, 2000; Zhao et al., 2018), although, the upstream death receptors activators were not examined in this study. Taken together, we provide evidence of overactive apoptotic signalling within the villous placenta that may lead to preterm birth (Figure 5). However, we found no correlation between the level of placental apoptosis and baby birth weight, suggesting that augmented placental apoptosis is more likely linked with mechanistic pathways of preterm labour without affecting fetal growth in the context of spontaneous delivery.

The upstream mediators that enhanced apoptosis, increased BAX or suppressed BCL2 expression in preterm placenta in our study are uncertain. A previous study showed that the anti-inflammatory cytokine IL10 prevents apoptosis by upregulating BCL2, and this inhibitory action can be reversed by IL10 neutralising antibody (Levy & Brouet, 1994). Rituximab-mediated downregulation of IL10 suppresses BCL2 expression and enhances apoptosis in tumour cells (Alas et al., 2001). In addition, IL10 inhibits TNF-α-induced apoptosis (extrinsic pathway) in rat cardiomyocytes mediated by Akt via activation of STAT3 (Dhingra et al., 2011). In our study, *IL10* was significantly downregulated in preterm placenta. The downregulation of IL10 may lead to downregulation of BCL2 in the preterm placenta that we observed (Figure 5). Furthermore, *STAT3* gene was significantly downregulated in preterm placentas compared to the term group in our study (*p* = 0.001, Supplementary Figure S2). Therefore, the simultaneous downregulation of IL10 and STAT3 may contribute to TNF-α mediated apoptosis in trophoblast cells in villous placenta.

Pro-inflammatory cytokines induce apoptosis in various cell types (Rath & Aggarwal, 1999; Wang et al., 2008). We investigated gene expression profiles of a panel of pro-inflammatory Th1 cytokines including *TNF-ɑ*, *IFN-γ*, *IL1β* and *IL2* in our placenta samples. The gene expressions of these cytokines were not different in term and preterm groups. However, when we conducted a correlation analysis between Th1 vs. Th2 cytokines, we noted that a Th2 cytokine *IL4* had a significant positive correlation with *TNF-α* in term placenta, but this correlation was abolished in preterm placenta. Naturally, *IL4* and *TNF-α* are co-expressed genes. Abrogated IL4/TNF-α correlation likely can alter, at least in part, the Th1/Th2 balance leading to Th1 predominant immune responses. This pro-inflammatory tissue micro-environment may further favour apoptosis in trophoblasts in preterm placenta.

Macrophages and T-cells are the major sources of Th1 and Th2 cytokines in the placenta (Houser et al., 2011; Tilburgs et al., 2015). Villous placenta is predominantly populated with placental macrophages (Hofbauer cells) and non-Hofbauer cells, including CD8^+^ T-lymphocytes, dendritic and natural killer cells (Pereira, 2018; Pique-Regi et al., 2019; Swieboda et al., 2020; Toothaker et al., 2021). Therefore, the sources of our identified Th1 and Th2 cytokines were predominantly from Hofbauer cells as well as non-Hofbauer villous immune cells (as the maternal blood from the tissue was removed).

Macrophage functions are manifested by their state of polarity or activation, which are predominantly decided by the surrounding micromilieu (Brown et al., 2014). The paradigm of macrophage polarisation is vastly complex (Swieboda et al., 2020). Classically, M1 polarisation (Th1) of macrophages is promoted by TNF-α, IFN-γ, and TLR-4 agonists (Mantovani et al., 2004; Martinez & Gordon, 2014) and M2 polarisation (Th2) is mediated by IL4, IL10, IL13, IL33 and TGF-β (Mosser & Edwards, 2008; Kurowska-Stolarska et al., 2009; Martinez & Gordon, 2014). Placental macrophages (Hofbauer cells) follow this paradigm of activation and tend to remain as M2 phenotype to maintain pregnancy in health (Svensson et al., 2011; Kim et al., 2012; Brown et al., 2014; Zulu et al., 2019). In our study we found significant downregulation of two M2 polarising stimulators *IL10* and *IL13* in the preterm placenta. This altered micro-environment may affect normal Hofbauer cell function and deviate their dominant M2 polarised state to the M1 polarised state in the preterm placenta, which needs further validation by experimentation.

Apoptosis and autophagy are interconnected and remain at a balanced state to maintain tissue homeostasis (Pattingre et al., 2005; Mariño et al., 2014). Autophagy and apoptosis frequently occur in the same cell following a sequence in which autophagy precedes apoptosis (Mariño et al., 2014). Autophagy plays pivotal roles in trophoblast functions during normal placental development (Arikawa et al., 2012; Nakashima et al., 2013; Arikawa et al., 2016). There are three major types of autophagy: chaperone-mediated autophagy, microautophagy, and macroautophagy (referred to as autophagy throughout this paper) (Levine & Kroemer, 2008). Autophagy (macroautophagy) is a catabolic process that involves the sequestration of defective proteins and organelles within double-membrane autophagosomes, that subsequently fuse with lysosomes to form autolysosomes in which autophagic cargo is degraded (Mizushima & Komatsu, 2011). Starvation or oxidative stress triggers autophagy which is executed by a group of tightly regulated autophagy-associated genes (Mizushima & Komatsu, 2011). By forming a complex with class III phosphatidylinositol 3-kinase (PI3KC3), BECN1 plays a pivotal role in autophagosome formation. BCL2 binds with BECN1 and inhibits its function, and thus negatively regulates autophagy (Mariño et al., 2014). In our study, there was no difference in *BECN1* gene expression in term and preterm placentas.

Due to the downregulation of BCL2, we anticipated to observe increased autophagy in the villous tissue of preterm placenta. However, when we examined the autophagy flux by measuring p62 protein, we found a significantly higher level of this protein in the preterm placenta compared to the term placenta, indicating diminished autophagy in the preterm placentas. p62 is a ubiquitously expressed cellular protein and one of the best characterized substrates of selective autophagy. p62 directly interacts with LC3 (microtubule-associated protein light chain) and subsequently incorporated into the autophagosome, executing cargo degradation (Mizushima & Komatsu, 2011). Accumulation of p62 is accompanied with impaired autophagy, and therefore used as a marker of autophagy flux (Komatsu et al., 2007; Bjørkøy et al., 2009; Mizushima & Komatsu, 2011; Klionsky et al., 2016). p62 acts as a signalling hub that determine the cell fate whether they survive via activating the NF-kB pathway or undergo apoptosis via accumulating Caspase 8 and downstream effector caspases (Moscat & Diaz-Meco, 2009). In our study, effector Caspase 3 and Caspase 8 protein levels were significantly higher in the preterm placenta compared to term placenta. Therefore, it is plausible that reduced autophagy flux may have positive effects on increased apoptosis in villous tissue of preterm placenta that we observed (Figure 5).

The most likely causes of reduced autophagy in preterm placenta in our study were due to downregulation of *ATG7* and upregulation of Calpain 1 (CAPN1) which play crucial roles in autophagy. Calpain 1 (also known as µ-calpain) is one of the major ubiquitous intracellular Ca^2+^-activated non-lysosomal cysteine proteases family which is activated by micromolar concentration of calcium and plays crucial roles in the regulation of autophagy and apoptosis. The other member of calpain family is Calpain 2 (also known as m-calpain) which is activated by millimolar concentration of calcium. CAPN1 activation-mediated impaired autophagy flux has been implicated in various pathologies (Russo et al., 2011; Ong et al., 2019; Liu et al., 2021). Activation of CAPN1 cleaves autophagy regulators BECN1 and ATG5 which impairs autophagy flux, resulting in accumulation of autophagic substrates within the cells. This phenomenon can be reversed by inhibiting CAPN1 (Russo et al., 2011; Ong et al., 2019; Liu et al., 2021). Therefore, it is probable that elevated level of CAPN1 in preterm placenta could cleave autophagy-associated proteins and inhibit autophagy flux, and subsequently accumulate p62 protein (Figure 5). In fact, our analysis revealed a significantly positive corelation between CAPN1 and p62 proteins, which further support our presumption.

CAPN1-mediated cleavage of ATG5 switches from autophagy to apoptosis stimuli (Yousefi et al., 2006). CAPN1 cleaves ATG5 into amino-terminal cleavage products that translocate from cytosol to mitochondria, bind with anti-apoptotic protein BCL-XL (BCL2L1) and trigger cytochrome c release, caspase activation and subsequently apoptosis. This phenomenon is not cell-specific and therefore considered as a general feature of an apoptotic cell (Yousefi et al., 2006). Furthermore, CAPN1 cleaves BID, inducing cytochrome c release and apoptosis (Smith & Schnellmann, 2012). Calpain inhibition reduces mitochondrial fragmentation and abrogates cell deaths (Ong et al., 2019). It is therefore likely that increased level of CAPN1 inhibits autophagy flux, and at the same time promotes apoptosis in the preterm placenta (Figure 5).

On the other hand, ATG7 (an E1-like enzyme) is one of the early canonical autophagy genes that activates both ATG12-complex and LC3-complex systems simultaneously during maturation of autophagosomes (Mariño et al., 2014). Tissue specific deletion of *ATG7* gene in liver and brain in mice diminished local autophagy and accumulated p62 in cells (Komatsu et al., 2007; Inami et al., 2011). Selective deletion of *ATG7* in trophoblasts, but not the fetus, in mice significantly reduced the size of the placenta compared to the wild type, providing evidence of the importance of autophagy in placental development. Furthermore, the ATG7 knockout placentas were featured with shallow trophoblast invasion and defective vascular remodelling, both of which are the characteristics of the placenta in preeclampsia (Aoki et al., 2018). Accumulation of p62 protein in the extravillous trophoblast cells in preeclamptic placentas has been reported, suggesting an association of impaired autophagy in the pathogenesis of preeclampsia (Nakashima et al., 2013; Nakabayashi et al., 2016). Impaired autophagy via placental ATG7 disruption in mice has been shown to retard fetal development as well (Muralimanoharan et al., 2016).

There is a lack of evidence that dysregulated autophagy is involved in the pathogenesis of spontaneous PTB in human. However, studies employing a mouse model has shown that TLR-agonist-induced inflammation inhibits autophagy by downregulating *ATG4c* and *ATG7* genes in the placenta and uterus leading to preterm birth (Agrawal et al., 2015). Premature birth due to LPS-induced inflammation has also been reported in ATG16L1 knockout mice (Cao et al., 2016).

Here, we report impaired placental autophagy due to downregulation of the *ATG7* and upregulation of CAPN1 in villous placenta associated with spontaneous preterm birth in human (Figure 5). Furthermore, we show significant negative correlation between placental p62 protein level and neonatal birth weight, suggesting a detrimental effect of impaired placental autophagy on fetal growth. We speculate that, apart from downregulated ATG7, augmented apoptosis may suppress autophagy in villous tissue by degrading essential ATG proteins, particularly ATG3 (Oral et al., 2012) and BECN1 (Luo & Rubinsztein, 2010; Wirawan et al., 2010) proteins, by the cascade of active caspases that produced during apoptosis signalling. Interestingly, impaired autophagy aggregates p62 protein which activates CASP8 and enhances apoptosis (Mizushima & Komatsu, 2011), and thus can establish an autophagy-apoptosis vicious cycle within the tissue micromilieu which could be pathognomonic. In this autophagy-apoptosis vicious cycle the upregulated CAPN1 may play critical ‘dual-edge’ roles and provide a potential therapeutic target for spontaneous PTB (Figure 5). Further studies focusing on this putative autophagy-apoptosis link may open up novel therapeutic modalities, perhaps by exploiting autophagy agonists, for PTB. p62 is also worthwhile to further study as a biomarker candidate in maternal blood for prediction of PTB and monitoring intrauterine fetal growth.

### Study limitations

In this study we demonstrated the variations of apoptosis and autophagy in the preterm placenta compared to the term placenta. However, to confirm if our observations are truly related to spontaneous PTB and not just due to temporal differences in placental autophagy and apoptosis related to GA at the time of delivery, a comparison between GA-matched placenta samples would be required. However, it would be ethically and logistically impossible to compare preterm placentas from spontaneously labouring women to GA-matched preterm placentas obtained at planned caesarean section from non-labouring women who do not have any placental dysfunction disorders. Such a design would also be confounded by the potential impact of labour on studied placentas. Animal experimental models which match GA at study could provide additional insight not feasible through human studies.

However, previous studies on human placenta samples collected from different gestational stages showed no significant differences in autophagy flux in placentas between early, mid and late gestation with vaginal delivery (Hung et al., 2013) or between gestational ages from second trimester to term (Oh et al., 2008). In our study, autophagy flux was decreased in the preterm placenta suggesting an underpinning pathology. Furthermore, studies on human placentas showed that the level of apoptosis in villous trophoblasts increases along with gestational age, and highest level of apoptosis was observed in 39–41 weeks compared to 37–39 weeks in uncomplicated normal pregnancies (Athapathu et al., 2003) and post term pregnancies (Smith & Baker, 1999). Contrary to these findings, we detected increased apoptosis in preterm placentas compared to term placentas. Therefore, the impaired autophagy and augmented apoptosis that we observed in our preterm placentas are more likely to be due to spontaneous PTB, rather than merely due to variation in the gestational ages of study placentas.

## Conclusion

In this study, we examined the molecular status of and interaction between apoptosis, autophagy and inflammation within the identical tissue samples from each placenta taken from spontaneous term and preterm deliveries. We demonstrated impaired autophagy with augmented apoptosis due to downregulation of ATG7 with upregulation of CAPN1, and downregulation of BCL2 with upregulation of BAX respectively, in a passively Th1-biased inflammatory micro-environment of the villous placenta, which may lead to spontaneous PTB. However, it is important to note that our study provides evidence of aberrant autophagy and apoptosis and their mechanistic association with PTB, but do not establish their causal links. Further study is needed to better understand the role of the inflammation-autophagy-apoptosis axis in the pathogenesis of spontaneous PTB.

## Data Availability

The raw data of this study is stored at the University of Sheffield Research Data Depository and will be available from the corresponding author upon reasonable request.
