# Supplementary material for: Impaired autophagy with augmented apoptosis in a Th1/Th2-imbalanced placental micromilieu is associated with spontaneous preterm birth
Source: Front Mol Biosci. 2022 Aug 26;9:897228. doi: 10.3389/fmolb.2022.897228 (PMC9460763; doi:10.3389/fmolb.2022.897228)
Supplement: Supplementary file 1 [file DataSheet1.PDF]

Supplementary file: Akram et al 2022

Table S1: Primers for qRT-PCR. Optimised QuantiTect Primer Assay primers from Qiagen.

**Gene name**	**Ensembl Transcript ID**	**Gene Globe ID**	**Catalogue No**	**Company**
BAX	ENST00000293288	QT00031192	249900	Qiagen
BCL2	ENST00000360344	QT00025011	249900	Qiagen
BECN1	ENST00000246933	QT00004221	249900	Qiagen
ATG3	ENST00000283290	QT00069769	249900	Qiagen
ATG7	ENST00000354449	QT00008974	249900	Qiagen
ERVW-1	ENSG00000242950	QT00270480	249900	Qiagen
DYSF	ENST00000258104	QT00035749	249900	Qiagen
GAPDH	ENST00000229239	QT00079247	249900	Qiagen
IFNγ	ENST00000229135	QT00000525	249900	Qiagen
TNFα	ENST00000229681	QT00029162	249900	Qiagen
IL1β	ENST00000263341	QT00021385	249900	Qiagen
IL2	ENST00000226730	QT00015435	249900	Qiagen
IL4	ENST00000231449	QT00012565	249900	Qiagen
IL5	ENST00000231454	QT00001435	249900	Qiagen
IL10	ENST00000259152	QT00041685	249900	Qiagen
IL13	ENST00000304506	QT00000511	249900	Qiagen
STAT3	ENST00000264657	QT00068754	249900	Qiagen
CD86	ENST00000330540	QT00033915	249900	Qiagen
CDH1 (E-Cad)	ENST00000261769	QT00080143	249900	Qiagen
VIM	ENST00000224237	QT00095795	249900	Qiagen

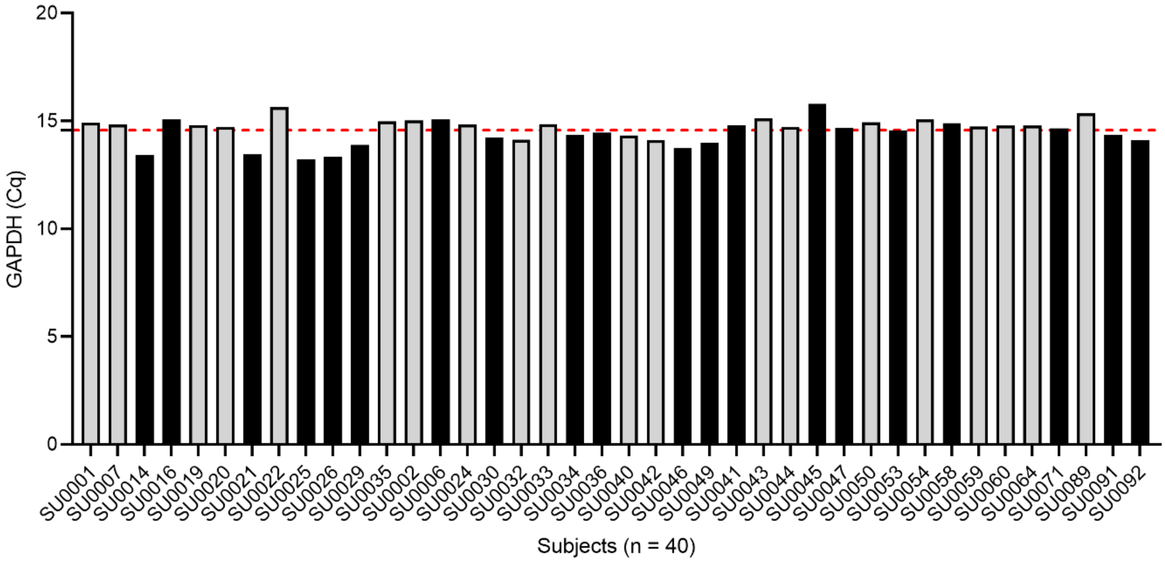

Figure S1: Cq values of GAPDH across the samples. Each bar represents the mean Cq from duplicate runs from each subject. Red dotted line represents the mean Cq of all 40 samples (14.57±0.09 SEM). Grey bars represent term placenta, black bars represent preterm placentas.

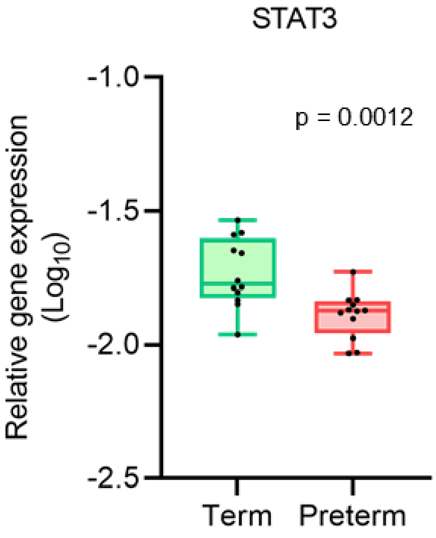

Figure S2: Box plots of qRT-PCR analysis showing gene expression (Relative to GAPDH) of STAT3 in term and preterm placentas. n = 12 term and 12 preterm. Data are presented as median and IQR with minima and maxima. Each dot represents individual subject. Mann-Whitney U test.
